# Th2-skewed T cells correlate with B cell response to **α**-Gal and tick antigens in **α**-Gal syndrome

**DOI:** 10.1172/JCI158357

**Published:** 2023-03-15

**Authors:** Danijela Apostolovic, Jeanette Grundström, Mensiena B. Gea Kiewiet, Marija Perusko, Carl Hamsten, Maria Starkhammar, Staffan Paulie, Marianne van Hage

**Affiliations:** 1 Division of Immunology and Allergy, Department of Medicine Solna, Karolinska Institutet and University Hospital, Solna, Sweden.; 2 Innovative Centre of the Faculty of Chemistry, Belgrade, Serbia.; 3 Department of Internal Medicine, Södersjukhuset, Stockholm, Sweden.; 4 Mabtech AB; Stockholm, Sweden.

**Keywords:** Immunology, Allergy, Th2 response

## Abstract

Tick bites have been shown to transmit a novel form of severe food allergy, the galactose-α-1,3-galactose (α-Gal) syndrome (AGS). Cellular responses to α-Gal in patients with AGS have, to date, not been thoroughly scrutinized. Therefore, we investigated T and B cell proliferation, activation, and cytokine profiles in response to tick protein extract (TE) and α-Gal-free TE in patients with AGS and in healthy controls. T and B cells from both patients and controls proliferated in response to TE, but significantly more in patients with AGS. B cell proliferation, but not T cell proliferation, in patients with AGS was reduced by removing α-Gal from the TE. In addition, TE induced a clear Th2 cytokine profile in patients with AGS. Expression of CD23 by B cells correlated only to T cell proliferation. However, both B cell proliferation and CD23 expression were reduced when CD40L and IL-4 were blocked. A large portion of the IgG1 and IgE antibodies binding TE in patients with AGS were directed against the α-Gal epitope. We have, for what we believe to be the first time, investigated T and B cell responses to α-Gal carrying tick proteins in patients with AGS, which will be essential for the understanding of the immune response against an allergenic carbohydrate transmitted by ticks.

## Introduction

Vector-borne zoonotic diseases are emerging at an increasing rate worldwide and are of major concern, largely due to changing global ecology (1). Among the most common pathogens transferred by tick bites are *Rickettsia ricketsii,* which causes Rocky Mountain spotted fever; Flaviviridae, including tick-borne encephalitis virus; and in the Northern hemisphere *Borrelia burgdorferi,* which causes Lyme disease (2). The pathogens are transferred to the host through the saliva, which contains a plethora of immunomodulatory proteins that facilitate tick attachment and feeding while simultaneously inducing an anti-tick immune response in the host (3). Recently, tick bites have been associated with a novel form of severe food allergy where IgE antibodies are formed against the carbohydrate epitope galactose-α-1,3-galactose (α-Gal) (4–6). Ticks from the genera *Amblyomma*, *Ixodes*, *Haemaphysalis,* and *Rhipicephalus* have been associated with induction of IgE to α-Gal (7).

The α-Gal epitope is present on glycoproteins and glycolipids from mammals, but it is not expressed in primates and old world monkeys due to evolutionary silencing of the α1,3 galactosyltransferase (GT) gene encoding the enzyme for α-Gal synthesis. It is suggested that pathogenic and nonpathogenic bacteria present in the human gastrointestinal flora are the cause of development of IgM and IgG antibodies against the α-Gal epitope (8, 9). IgE-mediated reactions against α-Gal–containing foods have become an emerging allergic disease globally. Patients experience immediate type allergic reactions 3–6 hours after ingesting mammalian meat, and the symptoms vary from urticaria and gastrointestinal problems to anaphylaxis (10, 11). Patients often require several visits to the emergency room before the link to mammalian meat consumption is discovered (12). In addition, patients can develop reactions to other products with a mammalian origin, e.g., gelatin-rich candies, dairy food products, therapeutic monoclonal antibodies, antivenom, and vaccines, which is why the disease is referred to as the α-Gal syndrome (AGS) (4, 5, 13, 14). We have previously shown that saliva from the *Ixodes ricinus* tick contains α-Gal–carrying proteins (15), and AGS is most likely induced by α-Gal transmitted to the host via bites. IgE levels to α-Gal have been shown to increase with the number of tick bites, and more than 1 tick bite was needed to induce IgE to α-Gal (16, 17), in contrast to Lyme disease, where a single bite can transmit disease (18).

Until now, there have been no data on the initiation of the allergic immune response to α-Gal in patients with AGS. In α1,3GT-knockout mice, T cells have been shown to be necessary for the induction of IgM antibodies to α-Gal (19). Moreover, subcutaneous sensitization of α1,3 GT-knockout mice with α-Gal conjugated to BSA in the presence of tick protein extract (TE) (20), or with tick salivary gland protein extract alone (21), leads to induction of α-Gal–specific IgE, strongly supporting the view that sensitization occurs via the skin, i.e., through tick bites. However, the involvement of human T cells in isotype switching and production of IgE antibodies in response to glycans, including α-Gal, still remains to be elucidated.

In the present study, we believe that we have, for the first time, investigated T and B cell responses against proteins from the European tick *I*. *ricinus* carrying the α-Gal epitope in patients with AGS. Our results provide a unique insight into the immune response against an allergenic carbohydrate transmitted by ticks.

## Results

In total, 50 patients with AGS and 19 individuals without AGS (controls) were enrolled in the study. All patients had IgE to α-Gal (median 18.5 kU_A_/L [range 0.76 to greater than 100 kU_A_/L]), and all except 1 patient reported being tick bitten. Most of the patients (41/49) also had IgE to *I*. *ricinus* TE (median 1.1 kU_A_/L [range < 0.1 – 14 kU_A_/L]). Of the 19 controls, 9 reported being tick bitten, but all were IgE negative to α-Gal and TE. There was no difference in the proportions of men and women between patients with AGS and controls (*P* = 0.43) or in which season the samples were collected (winter/spring or summer/autumn, *P* = 0.29), but the patients with AGS were significantly older than the controls (*P* = 0.008). For detailed characteristics of the patients, see Table 1 and of the controls see Table 2.

### CD4^+^ T cells from patients with AGS proliferate more in response to TE than CD4^+^ T cells from healthy controls.

**Figure 1:**
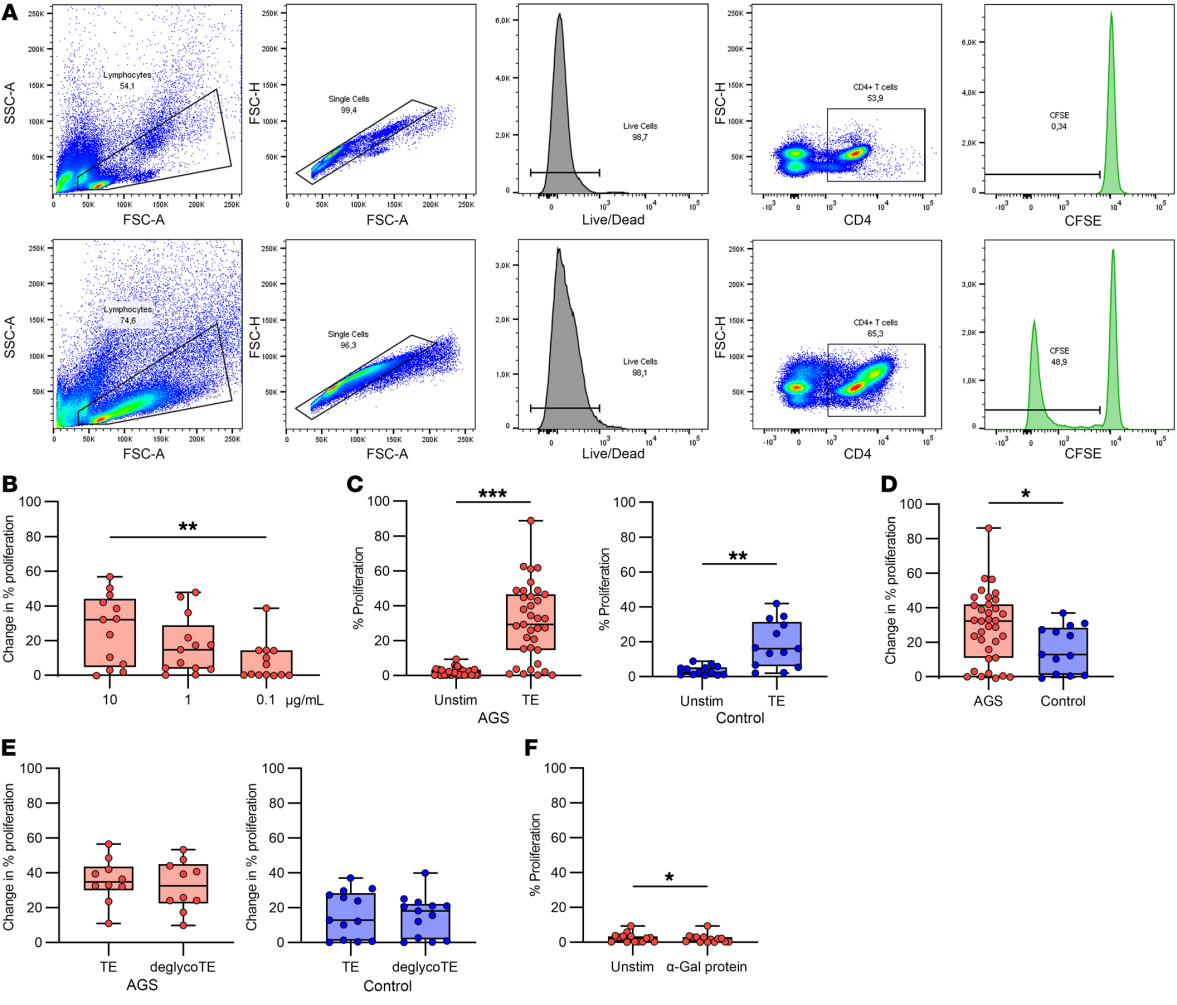
T cell proliferation measured by dilution of CFSE. (**A**) Gating strategy for proliferation of CD4^+^ T helper cells. (**B**) T cell proliferation in response to different doses of TE in patients with AGS, Friedman test with Dunn’s multiple comparisons test, **P < 0.01, *n* = 13. (**C**) T cell proliferation to TE compared with unstimulated cells in patients with AGS (left, *n* = 35) and healthy controls (right, *n* = 16), Wilcoxon matched-pairs signed rank test, **P < 0.01, ***P < 0.001. (**D**) Comparison of patients with AGS and healthy controls, Mann-Whitney U test, *P < 0.05, *n* = 35 (AGS) and *n* = 13 (controls). (**E**) T cell proliferation after removal of α-Gal from the TE in patients with AGS (left, *n* = 10) and healthy controls (right, *n* = 13), Wilcoxon matched-pairs signed rank test. (**F**) T cell proliferation to an α-Gal containing nontick protein in patients with AGS, Wilcoxon matched-pairs signed rank test, *P < 0.05, *n* = 14. Each point within the box plot represents 1 individual. Box plots represent IQR and median, whiskers extend to the farthest data points.

Peripheral blood mononuclear cells (PBMCs) labeled with carboxyfluorescein succinimidyl ester (CFSE) were cultured for 7 days in the presence of different stimulants for analysis of T cell proliferation. The gating strategy for proliferating T cells is depicted in Figure 1A. Analysis of CFSE dilution revealed that T cell proliferation in patients with AGS was dose-dependent, where 10 μg/mL TE gave the strongest response and was significantly higher than at 0.1 μg/mL (Figure 1B, *P* = 0.007, *n* = 13). T cells from patients with AGS and controls proliferated in response to TE (Figure 1C, *P* < 0.001, *n* = 35 [AGS]; *P* = 0.001, *n* = 13 [controls]), but there was significantly higher proliferation in patients with AGS compared with controls (Figure 1D, *P* = 0.043, *n* = 35 [AGS] and *n* = 13 [control]). Interestingly, there was no difference in the T cell proliferation between tick-bitten and nonbitten controls (Supplemental Figure 1; supplemental material available online with this article; https://doi.org/10.1172/JCI158357DS1). To test the α-Gal dependence of the T cell proliferation, the PBMCs were also stimulated with deglycosylated TE, in which the α-Gal epitope had been enzymatically removed by α-galactosidase treatment. There was no difference in proliferation of T cells to deglycosylated TE compared with unaltered TE (Figure 1E, *P* = 0.28, *n* = 10 [AGS]; *P* = 0.85, *n* = 13 [controls]). However, in 5 patients with AGS and 3 controls, the proliferation was reduced by more than 22%, whereas only 1 patient and 1 control showed an increased proliferation of more than 22%. Furthermore, there was a statistically significant decrease in proliferation of T cells from patients with AGS when stimulated with a nontick α-Gal–containing protein compared with unstimulated cells (Figure 1F, *P* = 0.04, *n* = 14). Still, the difference was less than 3% for all individuals, and the levels of proliferation were less than 6% for all data points. In addition, patients with AGS’s T cell proliferation did not correlate with IgE levels to α-Gal or TE (ρ = –0.187, *P* = 0.28, *n* = 35, and ρ = –0.257, *P* = 0.14, *n* = 35, respectively).

### Patients’ cytokine secreting cells are Th2-skewed after activation by TE.

**Figure 2:**
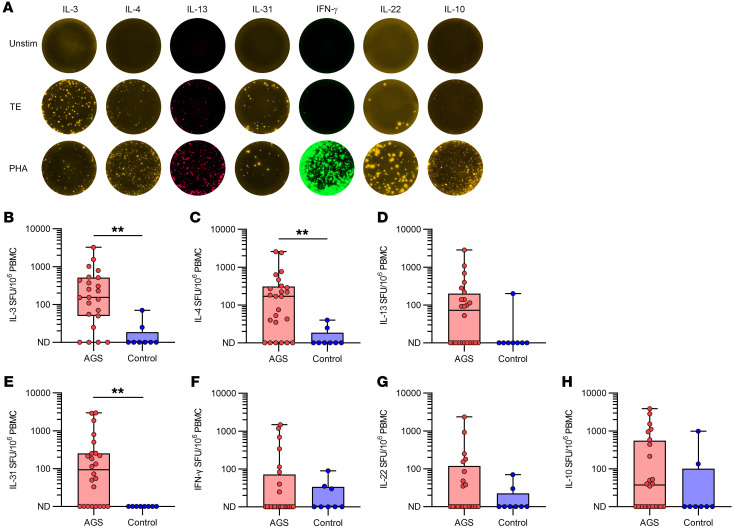
Cytokine expression by PBMCs in response to TE from patients with AGS and healthy controls. (**A**) Representative photos of FluoroSpot wells from a patient with AGS for unstimulated cells, TE-stimulated cells, and PHA-stimulated cells. (**B**) IL-3, (**C**) IL-4, (**D**) IL-13, (**E**) IL-31, (**F**) IFN-γ, (**G**) IL-22, and (**H**) IL-10. Mann-Whitney U test, **P < 0.01, *n* = 24 (AGS, *n* = 23 for IL-3) and *n* = 8 (control). Each point within the box plot represents 1 individual. Box plots represent IQR and median, whiskers extend to the farthest data points.

PBMCs were cultured for 40 hours together with TE in FluoroSpot plates to detect cells secreting specific cytokines. An example of IL-3, IL-4, IL-13, IL-31, IFN-γ, IL-10, and IL-22 secreting cells detected by FluoroSpot with unstimulated cells, TE- or phytohemagglutinin-stimulated (PHA-stimulated) cells from an AGS patient is depicted in Figure 2A. Overall, cytokine production was detected in most patients, whereas it was detected in only a few of the healthy controls. Patients (*n* = 24 unless otherwise stated) had a Th2 profile of secreted cytokines, where the number of IL-3, IL-4, and IL-31 secreting cells were significantly increased compared with controls (*n* = 8; Figure 2B, *P* = 0.001, *n* = 23; Figure 2C, *P* = 0.0027; and Figure 2E, *P* = 0.0026, respectively). In fact, IL-31 was not detectable in any of the controls. The number of IL-13 secreting cells was also increased in patients compared with controls, but not significantly (Figure 2D, *P* = 0.061). In contrast with what was observed for the Th2 cytokines, there was no difference between patients with AGS and healthy controls in the number of cells secreting the Th1 cytokine IFN-γ (Figure 2F, *P* = 0.91), the Th22 cytokine IL-22 (Figure 2G, *P* = 0.24, *n* = 21), and the Treg cytokine IL-10 (Figure 2H, *P* = 0.21). IL-5 and IL-17 secreting cells were not detectable in most individuals after TE stimulation. When the PBMCs were cultured in the presence of PHA as positive controls, the numbers of IL-3, IL-4, and IL-31 secreting cells were higher in patients with AGS than in healthy individuals (*P* = 0.05 [*n* = 23 for patients], *P* = 0.02 and *P* = 0.002, respectively).

**Figure 3:**
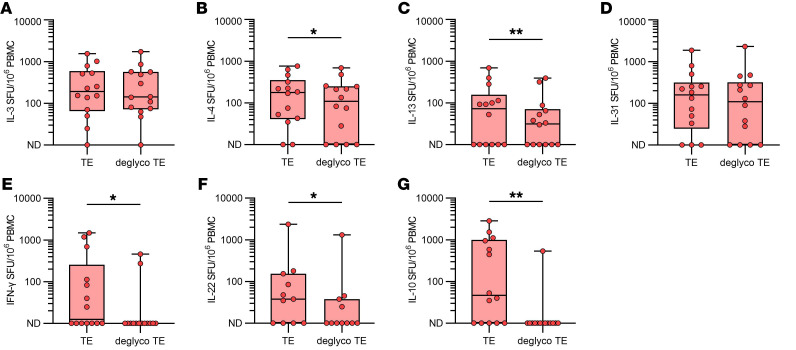
Cytokine secreting cells in PBMCs from patients with AGS after stimulation with TE and deglycosylated TE. (**A**) IL-3, (**B**) IL-4, (**C**) IL-13, (**D**) IL-31, (**E**) IFN-γ, (**F**) IL-22, and (**G**) IL-10. Wilcoxon matched-pairs signed rank test. *P < 0.05, and **P < 0.01. *n* = 14 for all except IL-22, where *n* = 11. Each point within the box plot represents 1 individual. Box plots represent IQR and median, whiskers extend to the farthest data points.

To investigate the effect of α-Gal on cytokine secretion, AGS patients’ cells were also stimulated with deglycosylated TE in the FluoroSpot assay (*n* = 14 unless otherwise stated). For IL-3 and IL-31 the number of cytokine secreting cells was unchanged for deglycosylated TE compared with unaltered TE (Figure 3A, *P* = 0.68, and Figure 3D, *P* = 0.20, respectively), whereas for IL-4 and IL-13 there was a significant decrease in the number of cytokine secreting cells (Figure 3B, *P* = 0.02, and Figure 3C, *P* = 0.008, respectively). When analyzing IFN-γ, IL-22, and IL-10, a substantial reduction in the number of cytokine secreting cells was noted for all of them (Figure 3E, *P* = 0.02, Figure 3F, *P* = 0.03, *n* = 11, and Figure 3G, *P* = 0.04, respectively).

### B cells highly express the activation marker CD23 after stimulation with TE.

**Figure 4:**
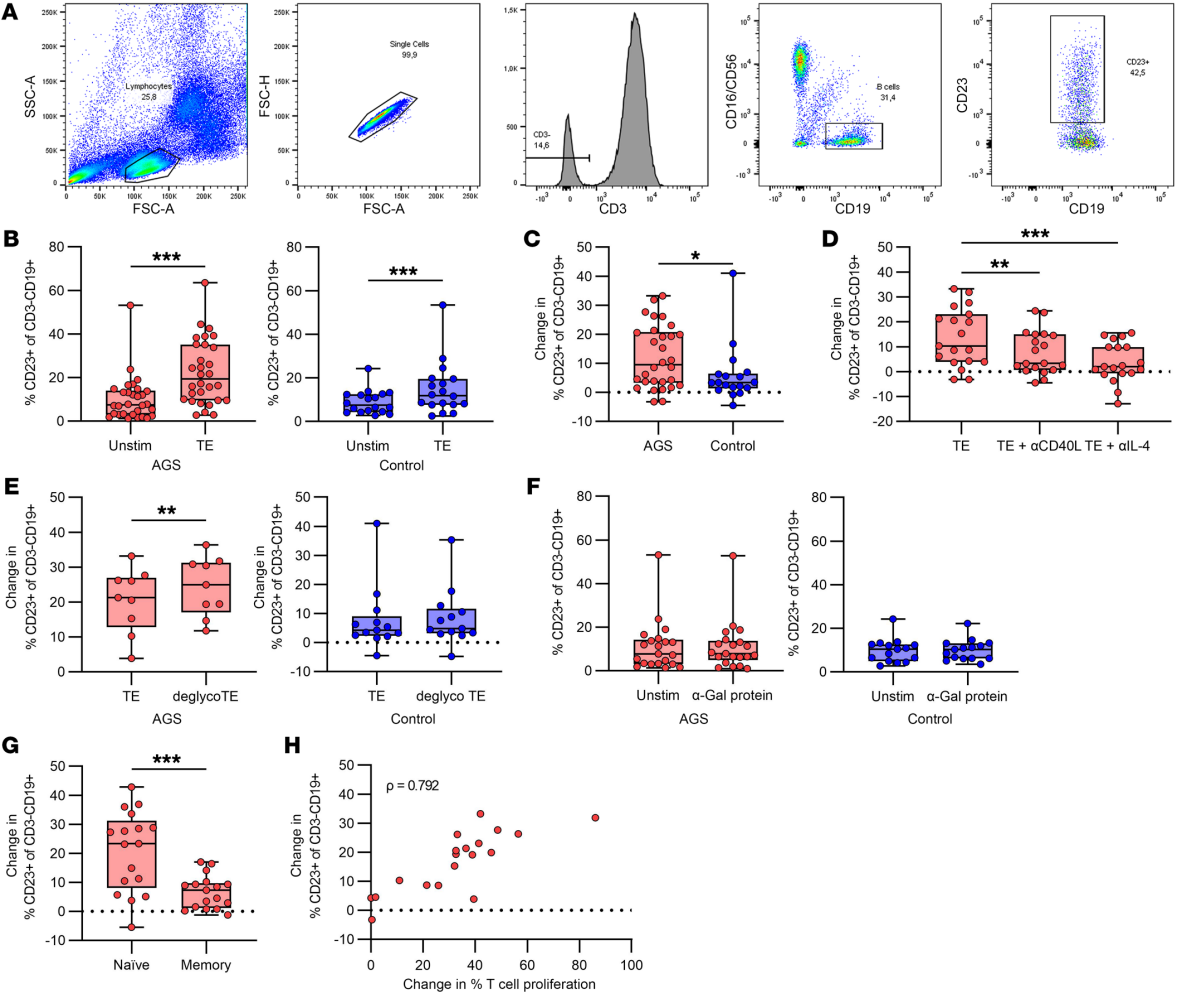
B cell expression of CD23. (**A**) Gating strategy for CD23-expressing B cells. (**B**) CD23 expression in unstimulated compared with TE stimulated B cells in patients with AGS (left, *n* = 30) and healthy controls (right, *n* = 18), Wilcoxon matched-pairs signed rank test, ***P < 0.001. (**C**) Comparison of patients with AGS and healthy controls, Mann-Whitney U test, *P < 0.05, *n* = 30 (AGS) and *n* = 18 (controls). (**D**) Effect of inhibition with anti-CD40L and anti-IL-4 antibodies in patients with AGS, Friedman test with Dunn’s multiple comparisons test, **P < 0.01, ***P < 0.001, *n* = 19. (**E**) Effect of removing α-Gal from the TE in patients with AGS (left, *n* = 9), and healthy controls (right, *n* = 13), Wilcoxon matched-pairs signed rank test, **P < 0.01. (**F**) Comparison of CD23 expression in unstimulated B cells compared with B cells stimulated with an α-Gal containing nontick protein in patients with AGS (left, *n* = 21) and healthy controls (right, *n* = 15). (**G**) Comparison of CD23 expression by naive (CD27-IgD^+^) and memory (CD27^+^) B cells after TE stimulation in patients with AGS, Wilcoxon matched-pairs signed rank test, ***P < 0.001, *n* = 17. Each point within the box plot represents 1 individual. Box plots represent IQR and median, whiskers extend to the farthest data points. (**H**) Correlation of T cell proliferation and CD23 expression in response to TE in patients with AGS, Spearman’s rank correlation, ρ = 0.792, *P* < 0.001, *n* = 19. Each point represents 1 individual.

The gating strategy for B cells expressing CD23 after PBMCs were cultured for 20 hours in the presence of different stimulants is depicted in Figure 4A. We found that B cells expressed higher levels of CD23 after PBMCs were stimulated with TE compared with unstimulated cells (Figure 4B, *P* < 0.001, *n* = 30 [AGS]; *P* < 0.001, *n* = 18 [control]), but the CD23 expression was significantly higher in patients with AGS than in healthy controls (Figure 4C, p =0.028, *n* = 30 [AGS] and *n* = 18 [control]). Further investigation of the CD23 response in patients with AGS showed that upregulation of CD23 by TE could be inhibited with anti-CD40L and anti-IL-4 antibodies (Figure 4D, *P* = 0.002 and *P* < 0.001, respectively, *n* = 19), but not with an isotype-matched antibody control (Supplemental Figure 2A, *P* = 0.81, *n* = 19). Stimulation with deglycosylated TE led to significantly increased CD23 expression compared with TE stimulation in patients with AGS, whereas CD23 expression did not change for the controls (Figure 4E, *P* = 0.0078, *n* = 9 [AGS]; *P* = 0.40, *n* = 13 [control]). Furthermore, stimulation with a nontick related protein carrying the α-Gal epitope did not affect CD23 expression compared with unstimulated cells (Figure 4F, *P* = 0.89, *n* = 21 [AGS]; *P* = 0.96, *n* = 15 [control]). The CD23 expression was significantly higher from naive cells compared with memory cells in patients with AGS, defined as CD27^+^ (memory) and CD27-IgD^+^ (naive), after stimulation with TE (Figure 4G, *P* < 0.001, *n* = 17). Moreover, the CD23 expression strongly correlated with T cell proliferation in patients with AGS (Figure 4H, ρ = 0.792, *P* < 0.001, *n* = 19). However, the CD23 expression did not correlate to the α-Gal or TE-specific IgE levels (ρ = 0.132, *P* = 0.49 and ρ = 0.010, *P* = 0.96, respectively, *n* = 30).

### B cell proliferation in response to TE stimulation is partly α-Gal specific in patients with AGS.

**Figure 5:**
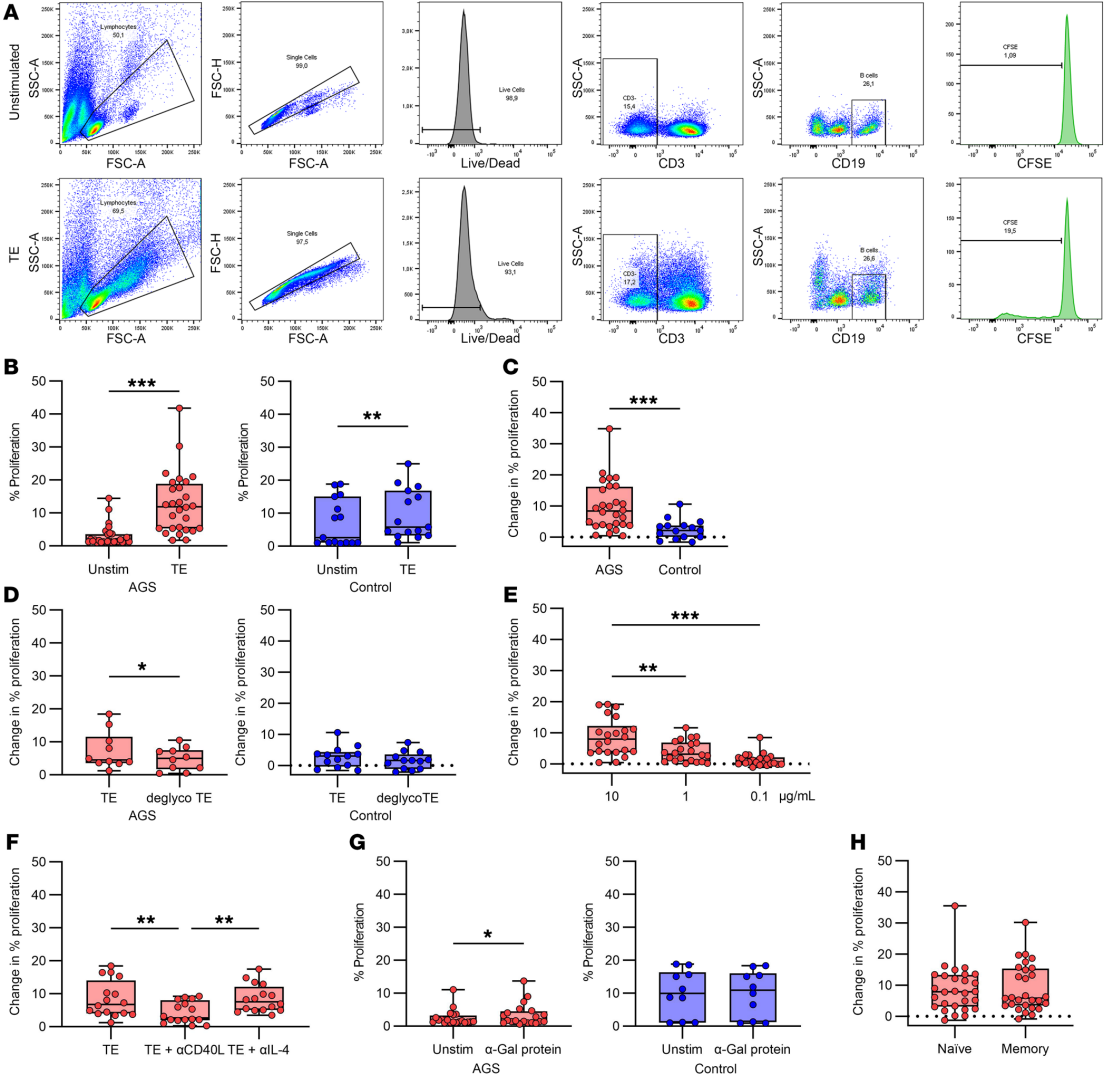
B cell proliferation measured by CFSE dilution. (**A**) Gating strategy for proliferation of CD3-CD19^+^ B cells. (**B**) Proliferation of unstimulated compared with TE stimulated B cells in patients with AGS (left, *n* = 28) and healthy controls (right, *n* = 15), Wilcoxon matched-pairs signed rank test, **P < 0.01 and ***P < 0.001. (**C**) Comparison of patients with AGS and individuals acting as healthy controls, Mann-Whitney U test, ***P < 0.001, *n* = 28 (AGS) and *n* = 15 (controls). (**D**) Effect of removing α-Gal from the TE in patients with AGS (left, *n* = 10) and individuals acting as healthy controls (right, *n* = 13), Wilcoxon matched-pairs signed rank test, *P < 0.05. (**E**) B cell proliferation in response to different doses of TE in patients with AGS, Friedman test with Dunn’s multiple comparisons test, **P < 0.01, ***P < 0.001, *n* = 22. (**F**) Effect of inhibition with anti-CD40L and anti-IL-4 antibodies in patients with AGS, Friedman test with Dunn’s multiple comparisons test, **P < 0.01, *n* = 16. (**G**) Comparison of B cell proliferation in unstimulated cells and cells stimulated with an α-Gal containing nontick protein in patients with AGS (left, *n* = 20) and individuals acting as healthy controls (right, *n* = 10), Wilcoxon matched-pairs signed rank test, *P < 0.05. (**H**) Comparison of proliferation in naive (CD27-IgD^+^) and memory (CD27^+^) B cells in patients with AGS, Wilcoxon matched-pairs signed rank test, *n* = 28). Each point within the box plot represents 1 individual. Box plots represent IQR and median, whiskers extend to the farthest data points.

Similar to T cells, CFSE dilution in B cells was analyzed after 5 days of culturing PBMCs in the presence of various stimulants. The gating strategy for proliferating B cells is depicted in Figure 5A. TE induced a significant proliferation in B cells from both patients with AGS and controls (Figure 5B, *P* < 0.001, *n* = 28, and *P* = 0.006, *n* = 15, respectively). However, the proliferation was significantly higher in patients with AGS compared with controls (Figure 5C, *P* < 0.001, *n* = 28 (AGS) and *n* = 15 (control)). B cells also proliferated in patients that did not show high CD23 expression. Furthermore, B cell proliferation in patients with AGS was significantly lower in response to deglycosylated TE compared with stimulation with unaltered TE (Figure 5D, *P* = 0.0137, *n* = 10), which was not observed in the controls (Figure 5D, *P* = 0.19, *n* = 13). B cell proliferation showed a similar dose dependency in patients with AGS as T cells did, with significantly reduced proliferation at the lower doses of TE (Figure 5E, *P* = 0.008 and *P* < 0.001 for 1 μg/mL and 0.1 μg/mL, respectively, *n* = 22). Proliferation in response to TE stimulation was inhibited by blocking with anti-CD40L, but not anti-IL-4 antibodies (Figure 5F, *P* = 0.002 and *P* > 0.99, respectively, *n* = 16) or with an isotype-matched antibody control (Supplemental Figure 2B, *P* = 0.11, *n* = 12). B cells from patients with AGS also showed a significant proliferative response to a nontick protein containing α-Gal, but the increase was less than 4 % (Figure 5G, *P* = 0.04, *n* = 20), whereas no proliferation was seen for the controls (Figure 5G, *P* = 0.75, *n* = 10). There was no difference in the proliferation of naive and memory B cells in response to TE in patients with AGS (Figure 5H, *P* = 0.94, *n* = 28). Furthermore, the B cell proliferation did not correlate with the IgE levels to α-Gal or TE (ρ = 0.306, *P* = 0.11, *n* = 28 and ρ = 0.177, *P* = 0.37, *n* = 28, respectively), or with T cell proliferation or CD23 expression (Supplemental Figure 3, ρ = 0.05, *P* = 0.84, *n* = 18 and ρ = 0.143, *P* = 0.48, *n* = 27, respectively).

### Patients with AGS’s antibody reactivity to TE is dominated by antibodies against α-Gal.

**Figure 6:**
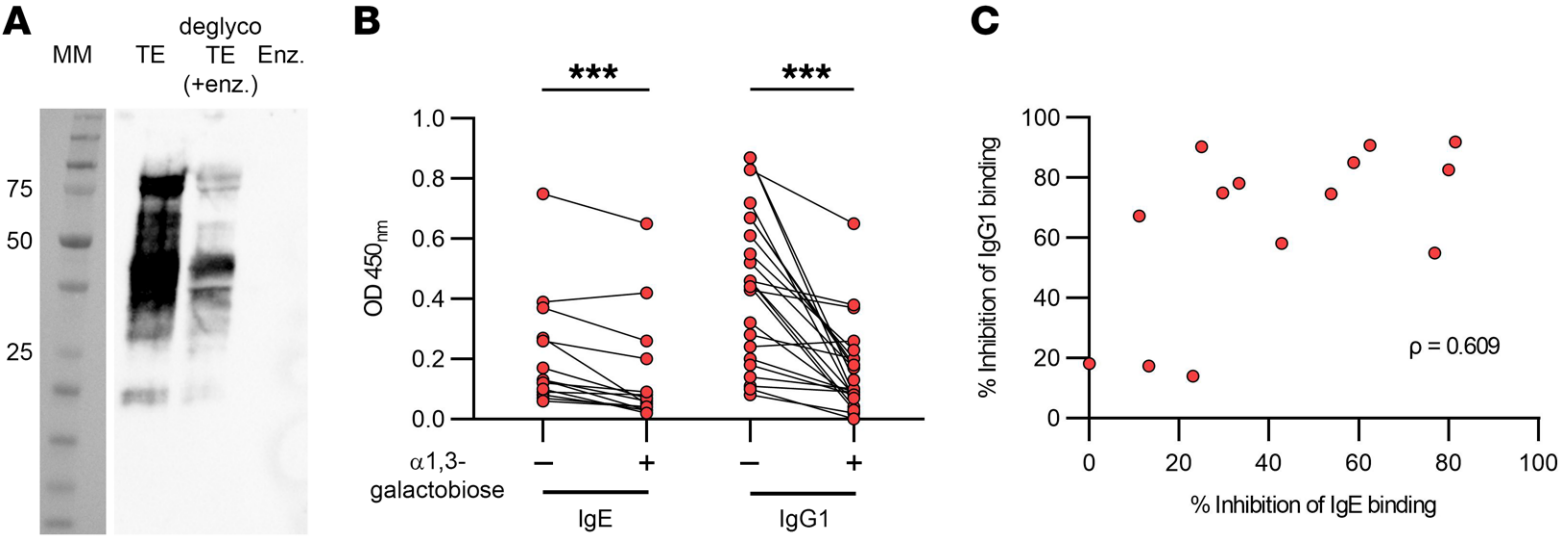
Patient antibody responses to TE. (**A**) Binding of a serum pool sample from a patient with AGS (*n* = 8) in a Western blot. MM = molecular marker, TE = tick extract, deglycoTE = deglycosylated TE, Enz. = α-galactosidase from green coffee bean. (**B**) Inhibition of IgE and IgG1 antibody binding to tick extract by α-Gal in patients with AGS. Wilcoxon matched-pairs signed-rank test, ***P < 0.001, for IgE *n* = 14, for IgG1 *n* = 23. Each pair of points connected by a line represents 1 individual. (**C**) Correlation of the inhibition of IgE and IgG1 antibody binding to TE by α-Gal. Spearman’s correlation coefficient ρ = 0.609, *P* < 0.05, *n* = 14. Each point represents 1 individual.

To assess to what extent the patients with AGS’s IgE reactivity to TE was directed toward α-Gal, Western blots were performed to detect IgE binding to TE and deglycosylated TE. IgE antibodies from a pool of AGS patient sera bound strongly to TE, whereas a much weaker binding to deglycosylated TE was observed (Figure 6A). The serum of a healthy control did not react with TE or deglycosylated TE (Supplemental Figure 4).

We also investigated the proportion of the IgG1 and IgE reactivity to TE that could be blocked by α-Gal in an inhibition ELISA. IgE binding to TE was detected in 14 of 24 patients with AGS tested, and the binding was inhibited up to 80% (median 38.1 %, range 0.0–81.5 %, Figure 6B, *P* < 0.001) by α-Gal disaccharide. IgG1 binding to TE was detected in 23 of 24 patients with AGS tested and the α-Gal disaccharide inhibited the response up to 100% (median 70.2 %, range 0.0–100.0 %, Fig. 6B, *P* < 0.001). Two of 9 tested controls had detectable IgG1 levels to TE, but these were lower than the levels detected in patients and were inhibited 6.3 % and 58.9 %, respectively, by α-Gal disaccharide. IgE levels to TE could not be detected in the controls, in line with the inclusion criteria. There was a moderate correlation between the inhibition of IgG1 and IgE binding in patients with AGS (Figure 6C, ρ = 0.609, *P* = 0.02, *n* = 14).

## Discussion

In contrast to preexisting anti-α-Gal responses — where natural production of IgM and IgG antibodies occur due to chronic exposure of B cells to the gut flora (22) — induction of IgE antibodies, and AGS, per se, is strongly correlated to and perpetuated by continuous tick bites (10). This is the only type of food allergy induced by tick bites, and until now, there have been no data on how the cellular responses to tick proteins are manifested in these patients. In this study, we have, for what we believe to be the first time, investigated patients with AGS’s T and B cell responses to TE. We found that TE induced a striking Th2-driven T cell response, which provided help in activation and proliferation of B cells. Notably, there was an α-Gal dependent component in B cell proliferation and in antibody binding to TE.

We found that T cells from patients with AGS proliferated after stimulation with TE and that this was not dependent on the presence of α-Gal, since there was no difference in comparison to the proliferation with α-Gal-deglycosylated TE. However, in 5 of 10 tested patients and 3 of 13 controls, the proliferation was substantially reduced after stimulation with deglycosylated TE, and, in those subjects, α-Gal on tick peptides was most likely important for the T cell response. Other studies have shown that there are T cells specific for α-Gal in humans (23), and that T cell proliferation in certain allergic individuals, e.g., venom-allergic individuals, can be dominated by glycan-specific clones (24). Similar to what was found for the patients with AGS, the T cells from healthy controls proliferated after stimulation with TE, but with significantly lower magnitude. Thus, the patients with AGS clearly have a stronger T cell response to TE. Earlier studies of allergen-specific T cell responses in patients with pollen allergies and healthy controls have shown that cells from both groups proliferate in response to the allergen (25, 26), but that the cytokine balance is different (26, 27).

In line with what has been previously shown for birch pollen (27), the cytokine profile in PBMCs activated by TE was clearly Th2-skewed for the AGS patients, which was not seen for the healthy controls. However, the number of cells secreting Th1, Th22, and Treg cytokines was not different between the 2 groups. We also found that the patients with AGS were more prone to produce Th2 cytokines in response to polyclonal stimulation with PHA than the healthy controls, which probably reflects the atopic background of the patients (11). Interestingly, salivary gland extract from *I*. *ricinus* ticks has been shown to induce Th2 cytokine production in human lymphocytes (28). Thus, the patients with AGS probably have tick protein–specific T cells that can initiate a secondary Th2 response in the immune reaction to new tick bites, which perpetuates the α-Gal allergy, whereas tick bitten non-AGS individuals show a more balanced response to TE. In addition, Hashizume and colleagues showed that the IgE levels to α-Gal increase and that the Th2/Th1 ratio of skin infiltrating T cells increases with the number of tick bites (16). Furthermore, we found that the number of Th2 cytokine secreting cells was unchanged or moderately decreased when the cells were stimulated with deglycosylated TE in patients with AGS. In contrast, Th1, Th22, and Treg cytokine producing cells were strongly decreased under the same stimulatory conditions. This indicates that the α-Gal in the TE induces a broad activation of T cells, in line with the general immunogenicity of the epitope that leads to induction of anti-α-Gal IgG in all immunocompetent humans (29). When the α-Gal is removed from the TE, the number of responding cells is reduced. The Th2-skewing component of the TE, however, seems not to be the α-Gal epitope, but rather the protein part of the TE, even though the result is production of α-Gal–specific IgE.

The low-affinity receptor for IgE, CD23, is upregulated on B cells after stimulation with IL-4 (30) and can be used as a marker of early activation. We found that B cells in patients with AGS and in healthy controls expressed increased levels of CD23 after stimulation with TE, but the increase was higher in patients with AGS. The expression of CD23 strongly correlated with T cell proliferation in patients with AGS, suggesting that T cells might have a part in the activation of these B cells. Indeed, we showed that the CD23 expression was dependent on T cells through IL-4 and CD40L. This finding is in line with previous reports on early activation of B cells showing the necessity of both IL-4 and contact with T cells activated through the T cell receptor (31). Furthermore, it has been shown that T cells from allergic donors upregulate CD40L and express IL-4 after short-term stimulation with allergen (26, 27). The unspecific nature of the CD23 expression was confirmed when the cells were stimulated with deglycosylated TE, and is likely due to soluble IL-4 produced by the T cells activated by TE. The cells expressing the highest levels of CD23 were naive B cells that have not encountered the allergen before, which agreed with previous studies (32, 33). Interestingly, it has been shown that upregulation of CD23 expression precedes the proliferation of B cells (34).

In patients with AGS, B cells strongly proliferated in response to stimulation with TE, which did not correlate with T cell proliferation. Earlier work by others have also shown the lack of correlation between T and B cell proliferative responses to allergens (35). Importantly, B cell proliferation is required to induce isotype switching and antibody production from both naive and memory B cells (36). B cells from the healthy controls also proliferated in response to TE, similar to what was found for T cells, but the proliferation was much lower than it was for the patients with AGS. This most likely reflects the presence of more TE-specific B cells in the patients with AGS. The B cell proliferation was reduced in patients with AGS when CD40L was blocked, suggesting that interaction with T cells promoted B cell proliferation. No effect of blocking IL-4 was seen. Importantly, there was also an α-Gal–specific component in the B cell activation to TE — since the proliferation decreased when α-Gal was removed — although it was not completely diminished. This, in accordance with the previous results, indicates that there is both an α-Gal specific and tick protein–specific component in the response to TE. Interestingly, previous results from WT mice showed that T cell help was needed for induction of tick-specific IgE, and that TE acted as an adjuvant for induction of α-Gal-specific IgE in the α1,3 GT knockout mouse (20). We noted that proliferation was initiated in both naive and memory B cells from patients with AGS, indicating a primary as well as a secondary type of response to the TE.

We could not detect any activation of B and T cells to nontick proteins containing α-Gal. Since the α-Gal epitope is a nonzwitterion carbohydrate, it cannot directly participate in MHCII priming. However, α-Gal specific IgE antibodies that are produced are specific for the α-Gal disaccharide (37). Furthermore, it is known that basophils are reactive both in vitro (38–41) and in vivo (42) to, for example, BSA-α-Gal or cetuximab, a reaction almost solely driven by IgE. Previous reports have shown that T cell clones from bee venom–allergic individuals can be specific for both the peptide and glycan epitope of phospholipase A2, in an MHCII restricted manner (24). Therefore, T cells in the sensitization and recall phases of AGS are most likely specific for tick proteins with or without α-Gal, but not for proteins from other sources even if they contain α-Gal. These T cells would promote activation of α-Gal–specific B cells presenting tick peptides on MHCII, and of bystander B cells in an α-Gal–independent manner. Interestingly, none of the analyzed cellular responses correlated with the IgE levels to α-Gal or TE. Thus, the T cell responses detected were independent of allergen-specific IgE antibodies. Earlier studies have also indicated the lack of correlation between T cell proliferation and IgE levels (25, 35).

A more thorough investigation of the TE-specific antibodies in patients with AGS and healthy controls showed that IgG1 antibodies were present in both groups, but IgE was only present in patients with AGS. However, IgE binding to TE could not be detected in all using ELISA, most likely due to their low IgE levels to TE as measured by ImmunoCAP. Both IgG1 and IgE binding to TE was inhibited by α-Gal. Western blot confirmed the results and showed strongly reduced binding of IgE in patients with AGS to deglycosylated TE. It was clear that a substantial part of the IgG1 and IgE responses in patients with AGS are toward the α-Gal component in the TE, but that there is also a response toward the protein component since removal of α-Gal from the TE could not completely abolish the response, in line with what we have previously shown (15).

A limitation of the study is that only the response to the water-soluble components of the TE — mainly the tick proteins — was investigated, and a possible response to α-Gal containing glycolipids might have been missed. However, in α1,3 GT knockout mice, sensitization with α-Gal glycolipids does not induce a strong antibody response, whereas α-Gal glycoproteins do (43). Another limitation is that some assays were based on few study subjects, due to the low number of cells obtained from some individuals. Unfortunately, we cannot exclude that nonbitten controls have been tick bitten without their notice, which can explain why some of the controls have an unexpected response in the T cell proliferation assay.

In conclusion, we found that T cells from patients with AGS are more strongly activated by TE compared with healthy controls, and show a Th2 response, where B cell activation at least partly depends on CD40L and IL-4. The B cell response is different, where B cells from patients with AGS proliferate to a greater extent than in healthy controls and the response is partially directed toward α-Gal. These results shed new light on the mechanisms of AGS and on the immune response to ticks.

## Methods

### Study design.

This study was designed to investigate T and B cell responses to TE in vitro and to measure the contribution of α-Gal in these responses. Peripheral blood was collected from the participants in the study, PBMCs were purified, and plasma and serum were separated. Different types of PBMC cultures were set up to analyze T and B cell proliferation and expression of CD23 by flow cytometry. Cytokine secreting cells were analyzed by FluoroSpot from frozen PBMCs. Investigation of the influence of the α-Gal epitope on antibody binding to TE was performed by inhibition ELISA and Western blot.

The study was a cross-sectional case-control study that was not blinded to the investigators. Patients with AGS and healthy controls were consecutively recruited to the different experiments. The number of patients included for each experiment is presented in the results and indicated in the figure legends.

### Patients and controls.

Fifty patients with AGS and 19 healthy controls were enrolled in the Stockholm area of Sweden from February 2017 to June 2022. The inclusion criteria for patients were individuals with a doctor’s diagnosis of AGS and IgE levels to α-Gal of more than 0.35 kU_A_/L. Healthy controls were IgE negative (< 0.1 kU_A_/L) to both α-Gal ImmunoCAP (code 0215; Phadia, Thermo Fisher Scientific) and *I*. *ricinus* TE measured by streptavidin ImmunoCAP, as previously described (5). Fifty-six percent (28 of 50 ) of the patients and 21 % (4 of 19 ) of the controls were atopic defined as having IgE greater than 0.1 kU_A_/L to birch pollen (t3), grass pollen (g6), the major cat allergen Fel d 1 (e94), or dust mites (d1). The Fel d 1 was used as proxy for cat allergies, since cat dander extract contains α-Gal. The seasons of sample collection were defined as winter, December to February; spring, March to May; summer, June to August; and fall, September to November. Both individuals acting as patients and controls answered a questionnaire about allergic symptoms and tick bites. All patients were seen by the same experienced physician with many years’ of diagnosing AGS. At this time point the patients answered the questionnaire, while controls answered the questionnaire upon inclusion in the study.

### Tick extract and removal of α-Gal from TE by deglycosylation.

Tick extract was prepared from adult *I*. *ricinus* female and male ticks (IS Insect Service GmbH) by crushing frozen ticks in PBS pH 7.4 using a Precellys tissue homogenizer (Bertin Technologies) and removing the solid pellet after centrifugation. For specific deglycosylation of the α-Gal epitope, TE was first buffer exchanged to PBS pH 6.6 using an Amicon Ultra 0.5 mL with 3 kDa cutoff (Millipore Sigma), and subsequently incubated with 2.5 U α-galactosidase from green coffee beans (Sigma-Aldrich) per mg of TE for 24 hours at 37°C. After incubation, the buffer was exchanged back to PBS pH 7.4 as before. Protein concentration was determined by bicinchoninic acid assay (Thermo Fisher Scientific) and endotoxin content was measured by limulus amebocyte lysate assay (Endosafe, Charles River) TE and deglycosylated TE contained less than 0.05 ng endotoxin/mg of protein. The lack of α-Gal in the deglycosylated TE was demonstrated by Western blot as previously described (44) and is presented in Supplemental Figure 5. Briefly, 10 μg of each protein extract was run under reducing conditions on an any kD mini-protean TGX precast gel (Bio-Rad). Proteins were blotted on to PVDF membranes (0.2 μm; Bio-Rad) using a Bio-Rad turbo system, and membranes were blocked overnight by incubation with blocking buffer (1% human serum albumin [HSA]; Sigma-Aldrich) in PBS with 0.05% Tween) at room temperature (RT). For detection of α-Gal containing proteins, the membranes were first incubated with primary chicken scFv anti-α-Gal labeled with a hemagglutinin (HA) tag, a gift from the Glycoscience group at the National University of Ireland (Galway, Ireland) (45), diluted 1:7,500, followed by 1 μg/mL secondary mouse-anti-HA-IgG (H3663, Sigma-Aldrich), and tertiary goat-anti-mouse-IgG labeled with alkaline phosphatase (115-055-146, Jackson Immunoresearch Laboratories) diluted 1:5,000, with washing in between. The presence of α-Gal was visualized by incubation with nitro blue tetrazolium/5-bromo-4-chloro-3-indolyl-phosphate (NBT-BCIP) alkaline phosphatase conjugate substrate kit (Bio-Rad Laboratories), followed by imaging on a Chemidoc instrument (Bio-Rad Laboratories).

### Setup of cell cultures.

PBMCs were separated from heparinized whole blood by density centrifugation over Ficoll (Cytiva). Aliquots of PBMCs were frozen at –80°C and stored in liquid nitrogen–cooled tanks until use in FluoroSpot. For detecting T and B cell proliferation, fresh PBMCs were resuspended in PBS at a concentration of 10 × 10^6^ cells/mL. CFSE (Invitrogen) was added to a final concentration of 1 pM, after which the cells were incubated for 15 minutes at 37°C. Cells were washed 3 times with 25 % autologous plasma in PBS and resuspended in serum-free AIM-V medium (Gibco) at a concentration of 1 × 10^6^ cells/mL and seeded in triplicate in flat-bottom 96-well plates (0.2 × 10^6^ cells/well). For analysis of CD23 expression on the surface of B cells, PBMCs were resuspended in complete RPMI 1640 medium (Hyclone) supplemented with 10% bovine growth serum (Gibco), 100 IU/mL penicillin (Cytiva), 100 μg/mL streptomycin (Cytiva), 2 mM L-glutamine (Cytiva), and 25 μg/mL gentamycin (Gibco) at a concentration of 1 × 10^6^ cells/mL. CFSE-labeled cells were stimulated with different doses of TE, 10 μg/mL, 1 μg/mL and 0.1 μg/mL, and the conclusion from these tests was that 10 μg/mL was optimal. In all cell experiments, PBMCs were stimulated with 10 μg/mL of TE; deglycosylated TE; Cetuximab for CD23 expression (Merck)/BSA-α-Gal or HSA-α-Gal for T cell proliferation (Dextra Laboratories)/HSA-α-Gal for B cell proliferation; or were left unstimulated as a negative control. For analysis of B cells, PBMCs were additionally stimulated with 10 μg/mL TE in the presence of either 3 μg/mL anti-IL-4 (clone MP-25D2), 15 μg/mL anti-CD154 (clone 24-31) or 15 μg/mL mouse IgG1 isotype control antibody (mouse IgG1 isotype MO PC-21 for anti-IL-4, and rat IgG1 isotype clone RTK2071 for anti-CD154) (all from BioLegend). As positive controls, 10 μg/mL PHA (Sigma-Aldrich) was used for T cell activation, 1 μg/mL R848 in combination with 10 ng/mL IL-2 (both from Thermo Fisher Scientific) was used for B cell proliferation, and 10 ng/mL IL-4 (BioLegend) was used for CD23 expression. T cell proliferation was analyzed after 7 days of culture, B cell proliferation after 5 days of culture, and CD23 expression after 18–20 hours of incubation at 37°C in a humidified incubator with 5% CO_2_.

### Flow cytometry.

After culture, the cells were analyzed by flow cytometry for expression of CD23 or proliferation by dilution of CFSE. For detection of live cells, fixable viability dye e780 (Invitrogen) or live/dead Aqua stain (Molecular Probes) was used. Antibodies used were IgD-FITC and -BV421 (clone IA6-2), CD27-PE and -APC (clone M-T721), CD19-PE (clone HIB19), CD4-APC-Cy7 (clone RPA-T4), CD19-APC-H7 (clone SJ25C1), CD16-BV421 (clone 3G8), and CD56-BV421 (clone NCAM16.2) from BD Biosciences, and CD3-PerCP-Cy5.5 (clone HIT3a), CD23-APC (clone EBVCS-5), CD8-APC (clone HIT8a), and CD4-BV421 (clone RPA-T4) from BioLegend.

At least 10,000 live cells or 3,000 CD3-CD19^+^ events were collected on a FACSCanto II flow cytometer (BD Biosciences). Data were analyzed using FlowJo v 10 software (FlowJo Software for Windows).

### FluoroSpot assay for analysis of T cell cytokines.

FluoroSpot assay was performed using FluoroSpot kits from Mabtech according to the manufacturer’s instructions. Briefly, Multiscreen 96-well IPFL plates (MultiScreen HTS 0.45 μm, binding immobilon-FL membrane; Millipore-Sigma) were first activated with 40% ethanol. After washing with sterile PBS, 1.5 μg capture monoclonal antibodies were added to the wells either as single antibodies, IL-3(clone IL3A), IL-10 (Clone 12G8) and IL-31 (clone MT31/88), or as combinations of several antibodies, IL-4 (clone IL4-I) + IL-5 (clone TRFK5) + IL-13 (clone MT1318), and IL-17 (clone MT44.6) + IL-22 (clone MT12A3) + INF-γ (clone IDIK), and the plates were incubated overnight at 4°C. After washing, wells were saturated with AIM-V medium for 30 minutes at RT and after removing the AIM-V, 200,000 thawed PBMCs suspended in AIM-V were added to each well with or without stimulation with 10 μg/mL TE or deglycosylated TE. AIM-V medium alone was used as negative control and 10 μg/mL PHA was used as positive control. For PHA stimulation, 100,000 cells were added. All tests were run in duplicates. Cells were incubated for 40 hours in a humidified incubator at 37°C with 5% CO_2_. After washing, spots of secreted cytokines — each spot representing a cytokine secreting cell — were detected by incubation for 1 hour at RT with detection monoclonal antibodies labeled with biotin or the peptide tags BAM and WASP. After washing again, fluorescently conjugated anti-BAM-490, streptavidin-550, and anti-WASP-645 (FSP-011803-2, Mabtech) were added for 1 hour at RT. Plates were washed before adding 100 μl of Fluorescence enhancer II to each well for 15 minutes. After discarding the Fluorescence enhancer, plates were air-dried before reading and analysis in an IRIS FluoroSpot reader (Mabtech). For analysis, a detection limit of at least 5 spots more than background was set and adjusted to the number of spot forming units (SFU)/10^6^ PBMCs. This limit of detection was possible to use, as the background was very low, which is normally the case for unstimulated cells. All values that were below the detection limit were set to 0.

### Western blot of patient and control IgE antibody binding to deglycosylated TE.

A Western blot of a patient serum pool and a control serum was performed to analyze the binding patterns to TE and deglycosylated TE. 10 μg of each protein extract was run on SDS-PAGE and blotted on to PVDF membranes as described above. The membranes were blocked for 2 hours by incubation with blocking buffer at RT and then incubated overnight with either a patient serum pool of sera from 8 patients (patients 16, 17, 20, 30, 31, 41, 42, and 46), or serum from control C6, both diluted 5 times in blocking buffer, or with blocking buffer alone for the secondary antibody control. The serum pool for patients with AGS had an IgE level to α-Gal of 83 kU_A_/L and 5.2 kU_A_/L to TE. For detection of proteins bound by the patient and control serum IgE, the membranes were incubated with secondary mouse-anti-human-IgE conjugated with horseradish peroxidase (clone B3102E8, Abcam), diluted 1:2,500. The protein bands were visualized with luminol (GE Healthcare) and evaluated in the Chemidoc instrument.

### Inhibition ELISA of antibody binding to TE.

Half-area 96-well medium binding microtitre plates (Greiner bio-1) were coated overnight at 4°C with 0.5 μg TE/well, followed by blocking with blocking buffer for 2 hours at RT. To investigate the potential inhibition of antibody binding to α-Gal, sera from 24 patients with AGS and 9 healthy controls were preincubated with α1,3-galactobiose (Galα1-3Gal, G203, Dextra Laboratories) at a concentration of 500 μg/mL or with blocking buffer for 2 hours at RT prior to addition to the plate (final dilution of sera was 1:5 for IgE and 1:50 for IgG1). IgE binding was detected using mouse-anti-human-IgE antibody conjugated with HRP (1 hour at RT, 1:2,500; ab99806, Abcam) and IgG1 binding with mouse-anti-human-IgG1 (1 hour at RT, 1:1,000; 555868, BD Biosciences), followed by incubation with HRP-conjugated sheep-anti-mouse-IgG (1 hour at RT, 1:5,000; 515-035-071, Jackson Immunoresearch Laboratories). Binding was visualized by incubation with TMB substrate (BioLegend) for 15 minutes before stopping the reaction with 1 M sulfuric acid. The absorbance was measured at 450 nm. Inhibition of IgE and IgG1 binding was calculated as [100–(OD_450nm_ inhibitor × 100) /OD_450nm_ no inhibitor]. The results are expressed as percent inhibition of IgE or IgG1 binding of duplicate determinations with a deviation of under 5 %.

### Statistics.

Differences between the AGS group and controls were calculated by Mann-Whitney U test, differences between different stimulants within the groups were calculated by Friedman’s test with Dunn’s multiple comparisons test if more than 2 stimulants were compared and by Wilcoxon matched-pairs signed rank test if 2 stimulants were compared. The association between 2 variables was calculated with Spearman’s rank correlation test. Fisher’s exact test was used to compare proportions. A *P* value of less than 0.05 was considered significant.

### Study approval.

The Swedish Ethics Review authority approved the study (Etikprövningsmyndigheten) with permit numbers 2011/1604-31/2, 2014/847-32, 2016/1447-32, 2018/2483-32, and 2020-01686. The study was performed in accordance with the declaration of Helsinki and all participants gave their written informed consent.

## Author contributions

DA, JG, MBGK, and MVH conceptualized the study. DA, JG, MBGK, and MP developed the methodology. MS, SP, and MVH provided the resources. DA, JG, MBGK, and MP performed the experiments. DA, JG, and MBGK analyzed the results. DA and JG were responsible for visualization of the project. DA and MVH acquired funding for the project. SP and MVH supervised the project. DA, JG, and MBGK wrote the original draft of the manuscript. DA, JG, MBGK, MP, CH, MS, SP, and MVH reviewed and edited the manuscript.

## Supplementary Material

Supplemental data

## Figures and Tables

**Table 2:**
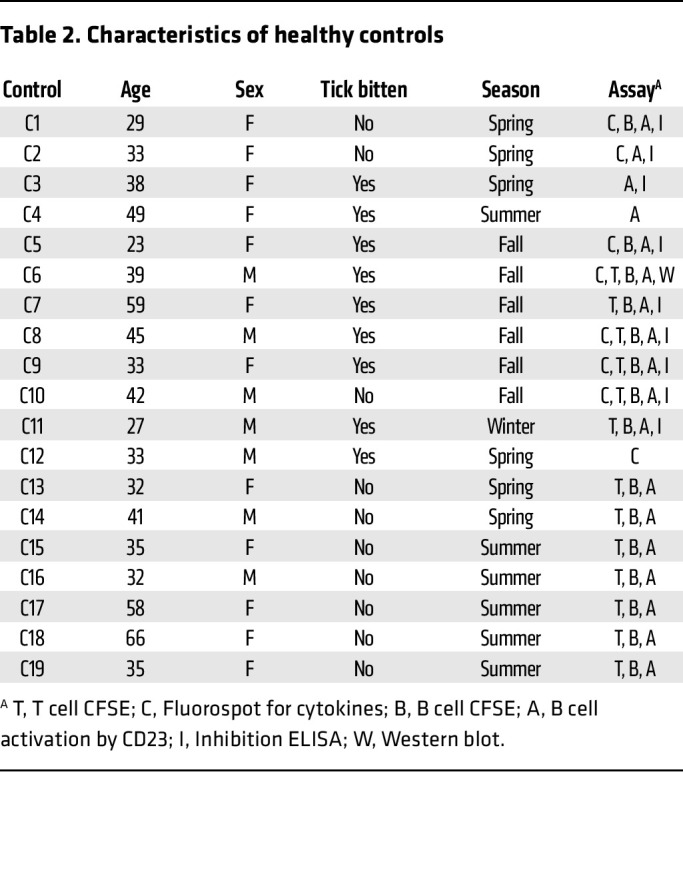
Characteristics of healthy controls

**Table 1:**
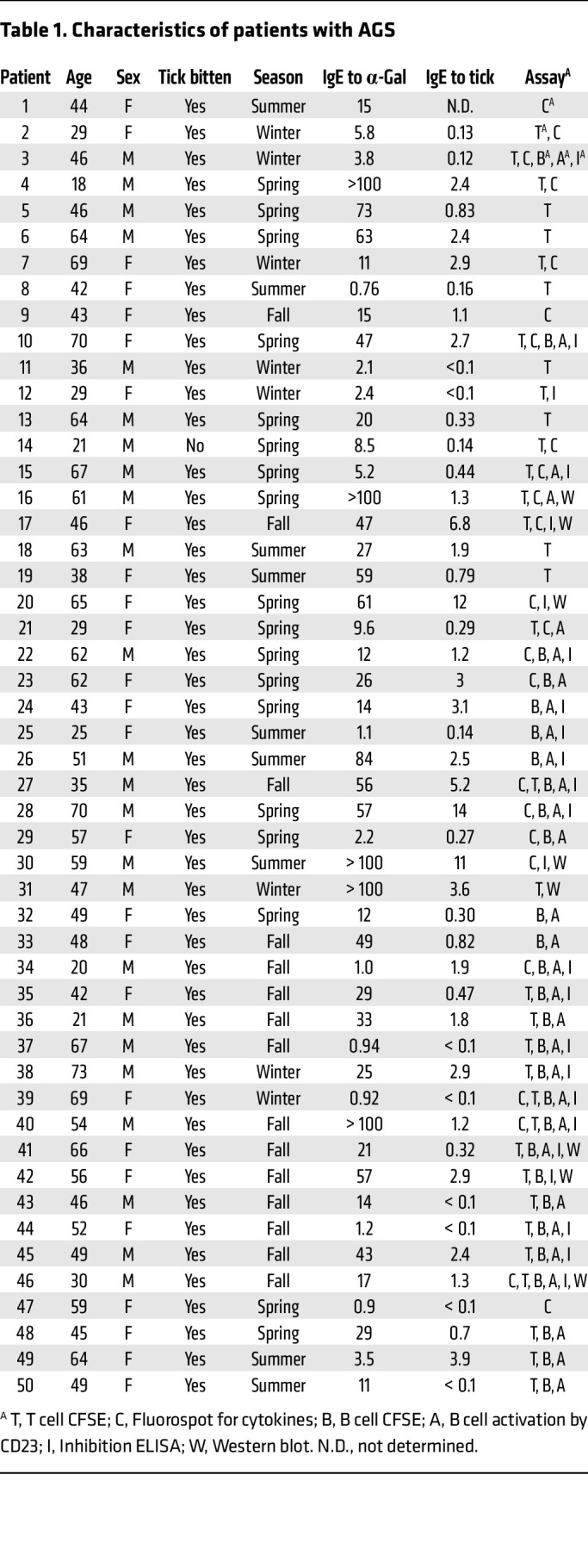
Characteristics of patients with AGS

## Version 1 01/26/2023

In-Press Preview

## Version 2 03/15/2023

Electronic publication

## References

[1] Swei A (2020). Patterns, drivers, and challenges of vector-borne disease emergence. Vector Borne Zoonotic Dis.

[2] Rochlin I, Toledo A (2020). Emerging tick-borne pathogens of public health importance: a mini-review. J Med Microbiol.

[3] Kitsou C (2021). Tick host immunity: vector immunomodulation and acquired tick resistance. Trends Immunol.

[4] Van Nunen SA (2009). An association between tick bite reactions and red meat allergy in humans. Med J Aust.

[5] Hamsten C (2013). Red meat allergy in Sweden: association with tick sensitization and B-negative blood groups. J Allergy Clin Immunol.

[6] Commins SP (2011). The relevance of tick bites to the production of IgE antibodies to the mammalian oligosaccharide galactose-α-1,3-galactose. J Allergy Clin Immunol.

[7] Cabezas-Cruz A (2019). Environmental and molecular drivers of the α-Gal syndrome. Front Immunol.

[8] Galili U (1988). Interaction between human natural anti-alpha-galactosyl immunoglobulin G and bacteria of the human flora. Infect Immun.

[9] Montassier E (2019). Distribution of bacterial α1,3-galactosyltransferase genes in the human gut microbiome. Front Immunol.

[10] Platts-Mills TAE (2020). Diagnosis and management of patients with the α-Gal syndrome. J Allergy Clin Immunol Pract.

[11] Kiewiet MBG (2020). Clinical and serological characterization of the α-Gal syndrome-importance of atopy for symptom severity in a European cohort. J Allergy Clin Immunol Pract.

[12] Houchens N (2021). Hunting for a diagnosis. N Engl J Med.

[13] Commins SP (2009). Delayed anaphylaxis, angioedema, or urticaria after consumption of red meat in patients with IgE antibodies specific for galactose-alpha-1,3-galactose. J Allergy Clin Immunol.

[14] Chung CH (2008). Cetuximab-induced anaphylaxis and IgE specific for galactose-alpha-1,3-galactose. N Engl J Med.

[15] Apostolovic D (2020). Allergenomics of the tick Ixodes ricinus reveals important α-Gal-carrying IgE-binding proteins in red meat allergy. Allergy.

[16] Hashizume H (2018). Repeated Amblyomma testudinarium tick bites are associated with increased galactose-α-1,3-galactose carbohydrate IgE antibody levels: a retrospective cohort study in a single institution. J Am Acad Dermatol.

[17] Mitchell CL (2020). Association between lone star tick bites and increased alpha-gal sensitization: evidence from a prospective cohort of outdoor workers. Parasit Vectors.

[18] https://www.cdc.gov/lyme/index.html.

[19] Cretin N (2002). The role of T cell help in the production of antibodies specific for Gal alpha 1-3Gal. J Immunol.

[20] Chandrasekhar JL (2019). Cutaneous exposure to clinically relevant lone star ticks promotes IgE production and hypersensitivity through CD4^+^ T cell- and MyD88-dependent pathways in mice. J Immunol.

[21] Choudhary SK (2021). Tick salivary gland extract induces alpha-gal syndrome in alpha-gal deficient mice. Immun Inflamm Dis.

[22] Chandrasekhar JL (2020). B cell responses in the development of mammalian meat allergy. Front Immunol.

[23] Wilhite T (2012). The effect of Gal expression on pig cells on the human T-cell xenoresponse. Xenotransplantation.

[24] Dudler T (1995). Carbohydrate-dependent, HLA class II-restricted, human T cell response to the bee venom allergen phospholipase A2 in allergic patients. Eur J Immunol.

[25] Wurtzen PA (1998). Dissection of the grass allergen-specific immune response in patients with allergies and control subjects: T-cell proliferation in patients does not correlate with specific serum IgE and skin reactivity. J Allergy Clin Immunol.

[26] Van Hemelen D (2011). Flow cytometric analysis of cytokine expression in short-term allergen-stimulated T cells mirrors the phenotype of proliferating T cells in long-term cultures. J Immunol Methods.

[27] Smith KA (2013). Characterisation of CD154+ T cells following ex vivo birch allergen stimulation defines a close relationship between T cell subsets in healthy volunteers. BMC Immunol.

[28] Kovar L (2001). Salivary gland extract from Ixodes ricinus tick polarizes the cytokine profile toward Th2 and suppresses proliferation of T lymphocytes in human PBMC culture. J Parasitol.

[29] Galili U (2013). Anti-Gal: an abundant human natural antibody of multiple pathogeneses and clinical benefits. Immunology.

[30] Bonnefoy JY (1995). CD23 and B-cell activation. Curr Opin Immunol.

[31] Crow MK (1989). Human peripheral blood T helper cell-induced B cell activation results in B cell surface expression of the CD23 (BLAST-2) antigen. Cell Immunol.

[32] Veneri D (2009). Expression of CD27 and CD23 on peripheral blood B lymphocytes in humans of different ages. Blood Transfus.

[33] Selb R (2017). CD23 surface density on B cells is associated with IgE levels and determines IgE-facilitated allergen uptake, as well as activation of allergen-specific T cells. J Allergy Clin Immunol.

[34] Fournier S (1994). Role for low-affinity receptor for IgE (CD23) in normal and leukemic B-cell proliferation. Blood.

[35] Eckl-Dorna J (2015). Poor association of allergen-specific antibody, T- and B-cell responses revealed with recombinant allergens and a CFSE dilution-based assay. Allergy.

[36] Akkaya M (2020). B cell memory: building two walls of protection against pathogens. Nat Rev Immunol.

[37] Plum M (2011). Close-up of the immunogenic α1,3-galactose epitope as defined by a monoclonal chimeric immunoglobulin E and human serum using saturation transfer difference (STD) NMR. J Biol Chem.

[38] Apostolovic D (2018). Immunoprofile of α-Gal- and B-antigen-specific responses differentiates red meat-allergic patients from healthy individuals. Allergy.

[39] Mehlich J (2019). The basophil activation test differentiates between patientswith alpha-gal syndrome and asymptomatic alpha-gal sensitization. J Allergy Clin Immunol.

[40] Michel S (2014). Skin prick test and basophil reactivity to cetuximab in patients with IgE to alpha-gal and allergy to red meat. Allergy.

[41] Hilger C (2016). Two galactose-α-1,3-galactose carrying peptidases from pork kidney mediate anaphylactogenic responses in delayed meat allergy. Allergy.

[42] O’Neil BH (2007). High incidence of cetuximab-related infusion reactions in Tennessee and North Carolina and the association with atopic history. J Clin Oncol.

[43] Tanemura M (2000). Differential immune responses to alpha-gal epitopes on xenografts and allografts: implications for accommodation in xenotransplantation. J Clin Invest.

[44] Perusko M (2021). Bovine γ-globulin, lactoferrin, and lactoperoxidase are relevant bovine milk allergens in patients with α-Gal syndrome. Allergy.

[45] Cunningham S (2013). Development of a convenient competitive ELISA for the detection of the free and protein-bound nonhuman galactosyl-α-(1,3)-galactose epitope based on highly specific chicken single-chain antibody variable-region fragments. Anal Chem.

