# Supplementary material for: Th2-skewed T cells correlate with B cell response to α-Gal and tick antigens in α-Gal syndrome
Source: J Clin Invest. 2023 Mar 15;133(6):e158357. doi: 10.1172/JCI158357 (PMC10014093; doi:10.1172/JCI158357)
Supplement: Supplemental data [file jci-133-158357-s038.pdf]

Supplemental Material

Th2-skewed T cells correlate with B cell response to α-Gal and tick antigens in α-Gal syndrome

Authors: Danijela Apostolovic,[1*] Jeanette Grundström,[1] M. B. Gea Kiewiet,[1] Marija Perusko,[1,2] Carl Hamsten,[1] Maria Starkhammar,[3] Staffan Paulie,[4] Marianne van Hage[1]

Affiliations:

[1]Division of Immunology and Allergy, Department of Medicine Solna, Karolinska Institutet and University Hospital; Solna, Sweden.

[2]University of Belgrade-Faculty of Chemistry, Innovative Centre, ltd; Belgrade, Serbia.

[3]Department of Internal Medicine, Södersjukhuset; Stockholm, Sweden.

[4]Mabtech AB; Stockholm, Sweden.

***Corresponding author:**

Danijela Apostolovic, PhD

Address: NKS BioClinicum J7:30, Immunology and Allergy Division, Visionsgatan 4, Solna SE17164, Sweden

Tel: +46 8 517 761 69

Email: danijela.apostolovic@ki.se

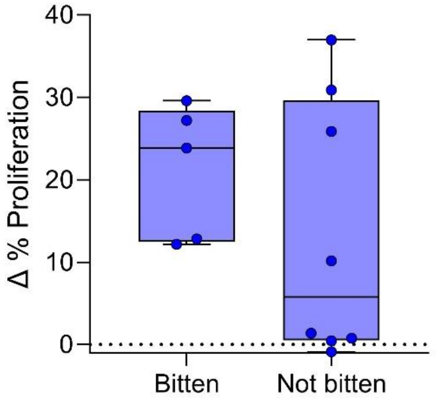

Fig. S1 T cell proliferation to tick extract of healthy controls stratified based on tick bites. Mann-Whitney U-test, p = 0.35, n = 5 (bitten) and n = 8 (non-bitten). Each point within the box plot represents one subject. Box plots represent IQR and median, whiskers extend to the farthest data points.

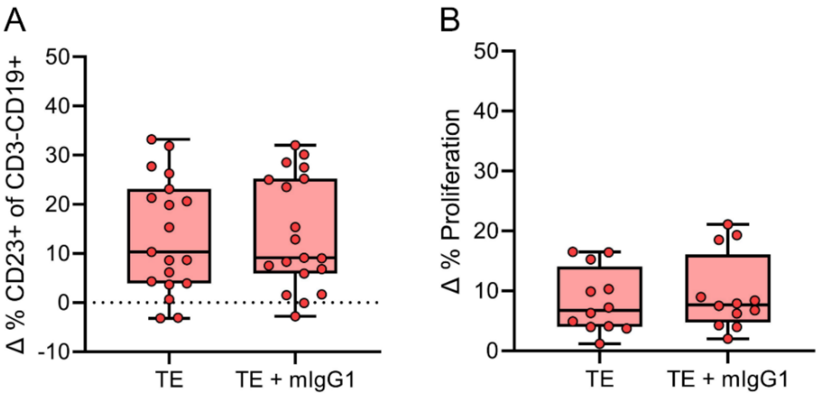

Fig. S2. Isotype control for blocking tick extract stimulation with anti-CD40L and anti-IL-4. (**A**) CD23 expression, n = 19, p = 0.81, and (**B**) proliferation by B cells, n = 12, p = 0.11. Wilcoxon matched-pairs signed rank test. Each point within the box plot represents one subject. Box plots represent IQR and median, whiskers extend to the farthest data points.

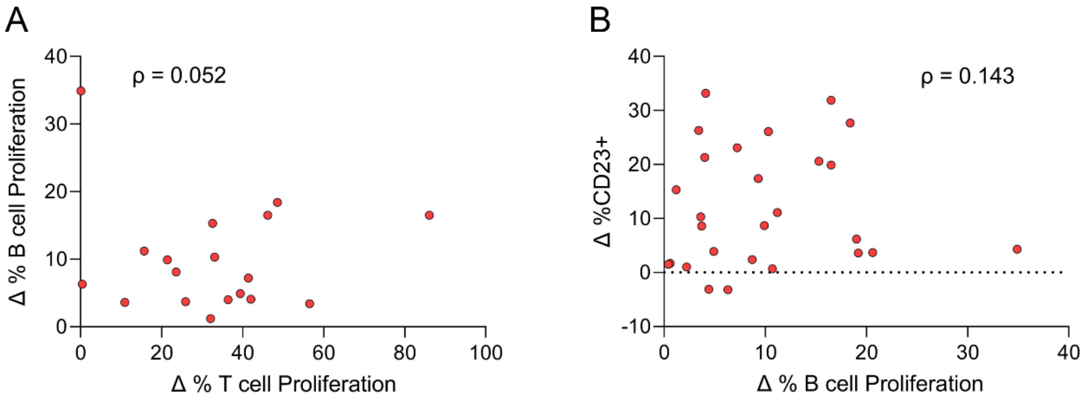

Fig. S2. Correlation between (**A**) B and T cell proliferation ρ = 0.052, p = 0.84, n = 18, and (**B**) CD23 expression and B cell proliferation ρ = 0.143, p = 0.48, n = 27. Spearman's rank correlation. Each point represents one subject.

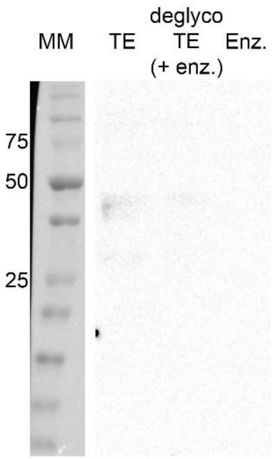

Fig. S3. Control serum binding to tick extract, deglycosylated tick extract and α-galactosidase in western blot.

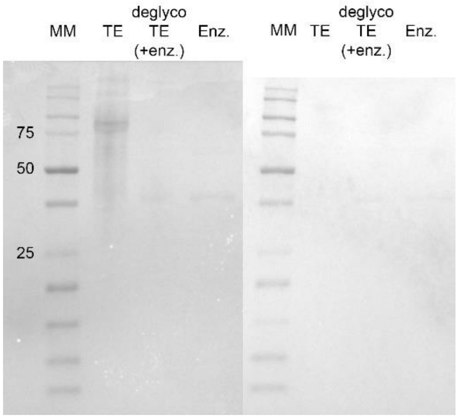

Fig. S4. Western blot for the presence of α-Gal in tick extract and deglycosylated tick extract. Left: chicken scFv-anti-α-Gal as primary antibody, right: antibody control without primary antibody. MM = molecular marker, TE = tick extract, deglycoTE = deglycosylated TE, Enz. = α-galactosidase from green coffee bean.
